# Characteristics, Whole-Genome Sequencing and Pathogenicity Analysis of *Escherichia coli* from a White Feather Broiler Farm

**DOI:** 10.3390/microorganisms11122939

**Published:** 2023-12-07

**Authors:** Shaopeng Wu, Lulu Cui, Yu Han, Fang Lin, Jiaqi Huang, Mengze Song, Zouran Lan, Shuhong Sun

**Affiliations:** 1 Shandong Provincial Key Laboratory of Animal Biotechnology and Disease Control and Prevention, College of Animal Science and Veterinary Medicine, Shandong Agricultural University, Tai’an 271000, China; 15662076726@163.com (S.W.); 2021010090@sdau.edu.cn (L.C.); 2021120549@sdau.edu.cn (Y.H.); 2018110416@sdau.edu.cn (F.L.); 2020110516@sdau.edu.cn (J.H.); songmz@sdau.edu.cn (M.S.); 2 Shandong Provincial Center for Animal Disease Control, Jinan 250000, China

**Keywords:** *Escherichia coli*, whole-genome sequencing, multidrug-resistant, pathogenicity, animal experiment

## Abstract

Avian colibacillosis, caused by avian *Escherichia coli* (*E. coli*), has historically been one of the most prevalent infectious diseases in large-scale poultry production, causing growth delays and mortality in chickens, resulting in huge economic losses. In recent years, the widespread use of antibiotics has led to the emergence of multidrug resistance in *E. coli* as a significant global problem and long-term challenge. Resistant *E. coli* can be transmitted to humans through animal products or the environment, which presents significant public health concerns and food safety issues. In this study, we analyzed the features of 135 *E. coli* strains obtained from a white feather broiler farm in Shandong, China, including antimicrobial susceptibility tests, detection of class 1 integrons, drug resistance genes, virulence genes, and phylogenetic subgroups. It is particularly worrying that all 135 *E. coli* strains were resistant to at least five antibiotic agents, and 100% of them were multidrug-resistant (MDR). Notably, the resistance genes of *bla*TEM, *bla*CTX-M, *qnr*S, *aaC4*, *tet*A, and *tet*B exhibited a high prevalence of carriage among the tested resistance genes. However, *mcr-2*~*mcr-9* were not detected, while the prevalence of *mcr-1* was found to be 2.96%. The most common virulence genes detected were *EAST1* (14.07%, encoding enterotoxins) and *fyu*A (14.81%, encoding biofilm formation). Phylogenetic subgroup analysis revealed that *E. coli* belonging to groups B2 and D, which are commonly associated with high virulence, constituted 2.22% and 11.11%, respectively. The positive rate of class 1 integrons was 31.1%. Whole-genome sequencing (WGS) and animal experiments were performed on a unique isolated strain called 21EC78 with an extremely strong membrane-forming capacity. The WGS results showed that 21EC78 carried 11 drug resistance genes and 16 virulence genes. Animal experiments showed that intraperitoneal injection with 2 × 10^5^ CFU could cause the death of one-day-old SPF chickens in 3 days. However, the mortality of Luhua chickens was comparatively lower than that of SPF chickens. This study reports the isolation of multidrug-resistant *E. coli* strains in poultry, which may pose a potential threat to human health via the food chain. Furthermore, the findings of this study enhance our comprehension of the frequency and characteristics of multidrug-resistant *E. coli* in poultry farms, emphasizing the urgent need for improved and effective continuous surveillance to control its dissemination.

## 1. Introduction

Gram-negative bacteria, such as *E. coli*, are important zoonotic pathogens [1]. The widespread use of antibiotics in the poultry industry has led to the emergence of widely resistant avian *E. coli* [2,3]. *E. coli* can enter the environment via animal feces and can spread through feedlot sheds, soil, air, and even water [4], while transmission through animals used in food production is another important route of *E. coli* transmission to humans [5,6,7].

The complexity and degree of resistance of *E. coli* strains vary widely between regions. Cross-infection with multiple drug-resistant bacteria carrying different drug resistance genes is a major challenge for the prevention and treatment process [8]. Hence, it is paramount to identify and characterize the resistance attributes of various drug-resistant strains. It is critical to scrutinize the resistance mechanisms and molecular characteristics of these avian *E. coli* strains in poultry farms to formulate a theoretical framework and novel protocols for clinical management and preventative measures. Virulence factors predominantly manifest as proteins encoded by genes located either in the chromosome or in plasmids. Consequently, more detailed research is warranted regarding the prevalence of virulence genes of *E. coli* isolates and their potential as repositories for antimicrobial resistance genes [9]. Class 1 integron is often reported in *E. coli* isolates recovered from various sources, including human, animal, and environmental samples. Their wide-ranging spread can be attributed to their binding to integrins and plasmids [10].

Biofilms are dynamic communities of heterogeneous microorganisms that transition from a free-swimming planktonic state to a sessile state embedded in the extracellular matrix [11]. Some antimicrobial resistance phenotypes and genes, particularly those conferring resistance to fluoroquinolones, have been reported to be associated with biofilm formation [12,13]. However, reports on the relationship between AMR and biofilms are inconsistent, suggesting that the true relationship is unclear [14,15].

In recent years, with the rapid development of genomics, whole-genome sequencing (WGS) technology has provided unique advantages in species identification, drug resistance and virulence prediction, genetic evolution analysis, and other aspects due to its rapid and high-resolution advantages [16]. The mapping of bacterial drug resistance and virulence genes by WGS is of great importance for the prevention and treatment of bacterial diseases by inferring the possible drug resistance and virulence phenotypes of bacteria [17,18].

The first objective of this study was to estimate antimicrobial resistance and the diversity, distribution of the resistance, and virulence genes in avian *E. coli* strains obtained from the white feather broiler farm. The second objective was to sequence the complete genome of the isolated *E. coli* with extremely strong membrane-forming ability and to evaluate its pathogenicity to different chicken breeds through different challenge methods. *E. coli* strains obtained from poultry farms will provide the foundation for *E. coli* epidemiology. Monitoring the resistance of *E. coli* is important to the assessment of potential economic and public health impacts. These data will provide a scientific basis for the clinical management and prevention of *E. coli* on farms in the region.

## 2. Materials and Methods

### 2.1. Information Regarding E. coli Strains

A total of 135 *E. coli* strains were isolated from a white feather broiler farm and stored at the Laboratory of Veterinary Public Health, Shandong Agricultural University. The isolation and identification of *E. coli* were performed as previously described [9,19].

### 2.2. E. coli Phylogrouping

The phylogroup of each strain was determined using the *E. coli* phylogrouping method described by Clermont et al. [20]. Briefly, this method assigns strains to phylogroups A, B1, B2, C, D, E, F, which belong to *E. coli* sensu stricto, while the eighth belongs to the Escherichia cryptic clade I. This methodology was developed using an extended quadruplex PCR and the multilocus sequence typing (MLST) scheme [21,22].

### 2.3. Antimicrobial Susceptibility Testing

All *E. coli* strains were assessed for resistance to 15 antimicrobial drugs’ susceptibility via the broth-diffusion method, according to the Clinical and Laboratory Standards Institute (CLSI) [23]. Briefly, the suspensions of bacterial were cultured and adjusted to the 0.5 McFarland turbidity standard, then using cotton swab to streak over the entire surface of Mueller–Hinton agar (Qingdao Hope Bio-Technology Co., Ltd., Qingdao, China). Then, the sensitabs were affixed to the plates and incubated at 37 °C for 16–18 h. Moreover, the diameter of the inhibition zone was measured with a ruler. The 15 antibiotics tested were ampicillin (AM, 10 μg), amoxicillin (AMX, 10 μg), trimethoprim-sulfamethoxazole (SXT, 25 μg), tetracycline (TET, 30 μg), meropenem (MEM, 10 μg), enrofloxacin (ENR, 15 μg), ceftriaxone (CTRX, 30 μg), doxycycline (DOX, 30 μg), cefotaxime (CTX, 30 μg), ceftazidime (CAZ, 30 μg), ofloxacin (OFX, 30 μg), amikacin (AMK, 30 μg), azithromycin (AZI, 30 μg), and sulfamonomethoxine (SMM, 30 μg). The antibiotic susceptibilities of polymyxin B were determined according to the microbroth-dilution assay [24]. *E. coli* strains resistant to more than three classes of antimicrobials were defined as multidrug-resistance (MDR) strains [25]. *E. coli* ATCC 25,922 was used as a quality control strain and was maintained at the Laboratory of Veterinary Public Health, Shandong Agricultural University [9].

### 2.4. Genomic DNA Extraction

The strains were propagated on 2 mL of LB and incubated at 37 °C in a shaking incubator for 10 h. Genomic DNA was extracted with the Genomic DNA Purification Kit (Tiangen Biotech, Beijing, China), and DNA templates were preserved at −20 °C prior to usage [9].

### 2.5. Detection of Class 1 Integrons and Drug Resistance Genes

PCR screening was applied for β-lactamase-encoding genes (*bla*CTX-M, *blaTEM*, *bla*SHV, *NDM*, *IMP*, *MIR*, *VIM*, *DHA*), genes associated with resistance to aminoglycosides (*aaC2*, *aaC4*), plasmid-mediated quinolone resistance genes (*qnr*C, *oqx*A, *qnr*S), tetracyclines (*tet*A, *tet*B, *tet*C), sulfonamides (*sul-1*, *sul-2*), polymyxins (*mcr-1*, *mcr-2*, *mcr-3*, *mcr-4*, *mcr-*5, *mcr-6*, *mcr-7*, *mcr-8*, *mcr-9*), and class 1 integron gene cassettes. All the primers and annealing temperatures were based on slight modifications from those previously described [26,27,28,29,30,31,32,33,34,35,36]. PCR products were analyzed via electrophoresis and observed on 1.5% agarose gels using ethidium bromide staining. Additionally, all PCR amplicons were sequenced to confirm gene identity.

### 2.6. Identification of Virulence Factors

The virulence genes encoding adhesin (*K99*, *987p*, *F17*, *F18*, *CS31A*), bundle-forming pilus (*bfp*A), shiga toxins (*Stx1*, *Stx2*), α-haemolysin (*hly*A), enterotoxins (*LT*, *Sta*, *EAST1*), yersiniatbactin biosynthesis (*irp2*), intimin (*eae*A), and iron scavenger receptor (*fyu*A) of *E. coli* were detected with the previously reported primers [37,38,39,40].

### 2.7. Biofilm Formation Assay

Biofilm formation was determined through a slight modification of the previously described method [41,42]. Briefly, the overnight cultures of *E. coli* strain were diluted to an OD_600_ of a thousand-fold in 3 mL of fresh LB broth, contained in polystyrene tubes at 37 °C with shaking for 12 h. After incubation, the growth medium was decanted, and the tubes were washed three times with sterile PBS buffer and air-dried. The biofilms were quantified using a crystal violet assay, with 250 μL of a 0.05% crystal violet solution being added to each well. The plates were incubated at room temperature for 15 min and rinsed with distilled water. The crystal violet was dissolved in 200 μL of 95% ethanol, and biofilm formation was analyzed at 570 nm.

### 2.8. Motility Assay

The freshly cultured bacterial solution was adjusted to an OD_600_ of 0.1, and 1 μL was punctured into the semi-solid LB solid medium at a depth of approximately 0.5 mm using a 10 μL pipette and then incubated in a constant temperature incubator at 37 °C for 4–24 h. The growth of the strain was observed, and the diameter of the strain was measured.

### 2.9. Whole-Genome Sequence Analysis

The WGS was performed with the Illumina Miseq System (Illumina, San Diego, CA, USA), and the paired-end Illumina reads were assembled by SPAdes v3.6.2. The preliminary annotation of draft genomes of all strains was performed using RAST (Rapid Annotation using Subsystem Technology) [43]. The CGE platform (http://www.genomicepidemiology.org/, accessed on 1 September 2022) was used for analyses of resistance genes (ResFinder 4.1; all antibiotic resistance databases were selected with a cut-off value of 95% identity and 80% minimum coverage) and virulence genes accessed on 1 September 2022. Plasmid replicons [44] and virulence-associated genes based on the *E. coli* virulence-associated gene databases EcVGDB and VFDB [45] were identified.

### 2.10. Lab Animal Experiment

The guidelines of the Institutional Animal Care and Use Committee (IACUC) of Shandong Agricultural University (Number: SDAUA-2022-21) were followed for the chicken experiment. One-day-old SPF chickens and Luhua chickens were purchased from Jinan SPAFAS Poultry Co. (Jinan, China) and Shandong Jinqiu Agriculture and Animal Husbandry Technology Co. (Jinan, China) The chickens were placed in a clean and tidy environment at around 32 °C, where they had unrestricted access to food and drinking water. The strains 21EC78 were cultured to the logarithmic growth phase, washed with PBS, and diluted to 2 × 10^5^–2 × 10^10^ CFU/mL.

### 2.11. Lab Animal Experiment 1: Differences in the Pathogenicity of Challenge Methods to SPF Chickens

Sixty one-day-old SPF chickens were randomly divided into ten groups. The challenge doses were 2 × 10^5^, 2 × 10^6^, and 2 × 10^7^ CFU/mL of the bacterial solution 100 μL for the intraperitoneal injection group and 2 × 10^5^, 2 × 10^6^, 2 × 10^7^, 2 × 10^8^, and 2 × 10^9^ CFU/mL of the bacterial solution 100 μL for the oral administration group. The control groups were treated with intraperitoneal injection or oral administration of PBS. The morbidity and mortality of chickens were observed daily for three days, and the number of dead chickens was recorded during the experiment to compare the differences in pathogenicity of the challenge methods on SPF chickens.

### 2.12. Lab Animal Experiment 2: Differences in the Pathogenicity to Chicken Breeds

Thirty-six one-day-old Luhua chickens were randomly divided into six groups. The concentration of bacterial solution was diluted into five gradients from 2 × 10^6^ to 2 × 10^10^ CFU/mL, and each chicken was inoculated with 100 μL of the bacterium by intraperitoneal injection. The control group was treated with intraperitoneal injection of PBS. The morbidity and mortality of the chickens were observed daily for three days, and the number of dead chickens was recorded during the experiment to compare differences in pathogenicity between chicken breeds.

### 2.13. Data Analysis

The statistical analyses of correlation matrix between resistance phenotype and genotype were performed using SPSS software version 21.0 (IBM Corp., Armonk, NY, USA), employing the chi-square test [46], where * and ** indicated a significant difference and * *p*  <  0.05 and ** *p*  <  0.01. The biofilm formation ability of *E. coli* strains was plotted using GraphPad Prism (Version 8.0.1) and expressed as mean ±95% confidence intervals of three replicates.

## 3. Results

### 3.1. Phylogrouping of E. coli Strains

According to the genomic similarity analysis using phylogrouping, *E. coli* from groups B2 and D, both of which are traditionally considered to be highly virulent, accounted for 2.22% and 11.11%, respectively, while *E. coli* from group B1 accounted for the most with 37.04% (Table 1).

### 3.2. Resistance Profiles of E. coli Strains

**Figure 1:**
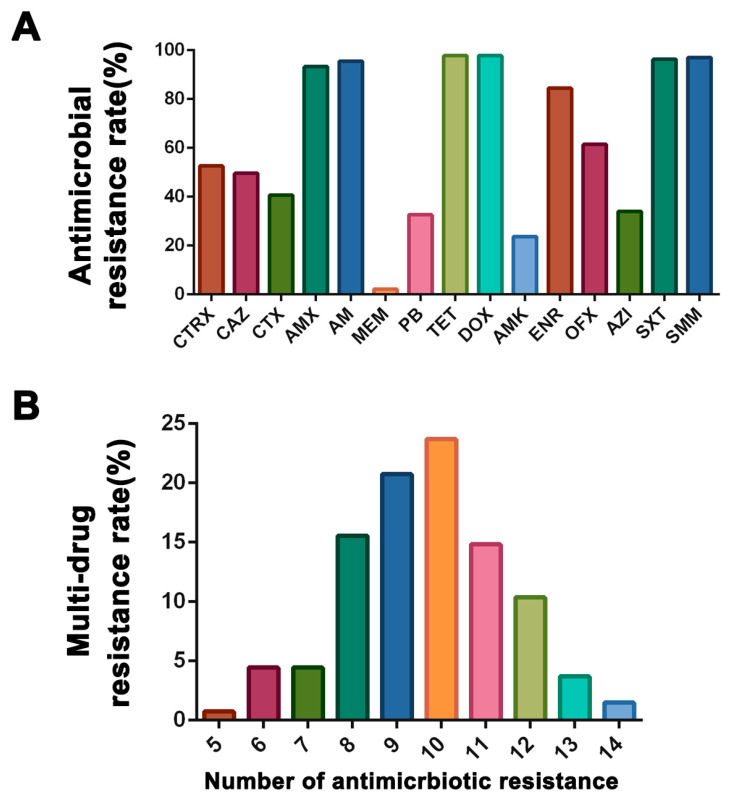
Analysis of antibiotic resistance in *E. coli* strains. (**A**) Rates of antibiotic resistance among *E. coli* strains. (**B**) Prevalence of multidrug resistance among *E. coli* strains. All the tested *E. coli* strains were resistant to at least five antibiotics.

The results of the antimicrobial susceptibility analysis of 135 *E. coli* strains differed in resistance to 15 antibiotics, as shown in Figure 1A. The resistance rates to AMX, AM, DOX, TET, SMM, and SXT were all above 80% in all the *E. coli* strains. In addition, all the tested *E. coli* strains were resistant to at least five antibiotics (Figure 1B). A total of 56 types of resistance profiles were found in the strains, of which CTRX-CAZ-CTX-AML-AM-TET-DOX-ENR-SXT-SMM was the most common (9.63%), followed by CTRX-CAZ-CTX-AML-AM-TET-DOX-SXT-SMM (8.89%) (Table 2). Notably, two of these strains were resistant to 14 kinds of the tested antibiotic categories, with the resistance profiles of CTRX-CAZ-CTX-AML-AM-TET-DOX-ENR-OFX-AMK-AZI-SXT-SMM-PB and CTRX-CAZ-CTX-AML-AM-MEM-TET-DOX-ENR-OFX-AMK-AZI-SXT-SMM (Table 2).

### 3.3. Detection of Antimicrobial Resistance Genes

**Figure 2:**
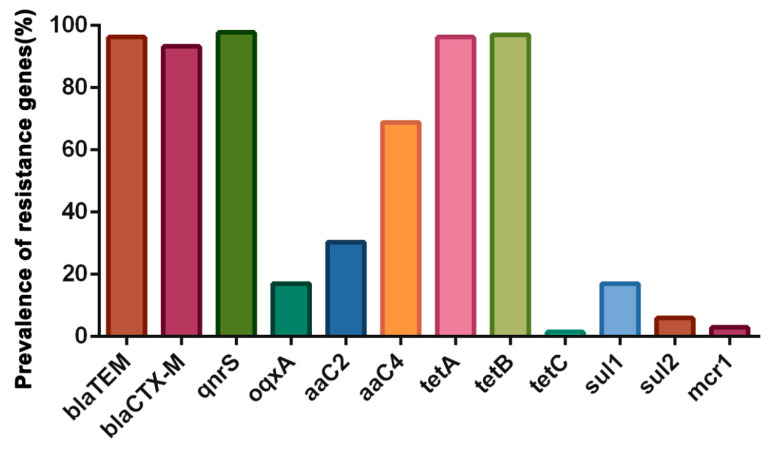
Prevalence of antimicrobial resistance genes in *E. coli* strains from different sample sources.

All the *E. coli* strains were analyzed for antimicrobial resistance genes, and the results are shown in Figure 2. The *bla*TEM (96.30%) and *bla*CTX-M (93.33%) were the most frequently strained β-lactamase genes, while *tet*A (96.30%) and *tet*B (97.03%) were the most frequently strained tetracycline resistance genes. In addition, three quinolone resistance genes were detected among the strains, and *qnr*S (97.78%) was the most commonly strained quinolone resistance gene. Two aminoglycoside resistance genes, *aaC4* (68.89%) and *aaC2* (30.37%), were also detected. However, *mcr2*-*mcr9* were not detected in this study, while *mcr-1* was carried by 2.96% of the analyzed strains.

### 3.4. Concordance of Genotypic–Phenotypic Antimicrobial Resistance

The concordance between genotypic and phenotypic resistance is delineated in Table 3. The correlation between genotypic and phenotypic antimicrobial resistance of the strains was scrutinized. Remarkably, a significant correlation was observed between the ceftriaxone resistance phenotype and *bla*CTX-M among the strains (*p*  = 0.004). Furthermore, the cefotaxime resistance phenotype and the cefradine resistance phenotype were significantly correlated with the *bla*CTX-M gene among the strains (*p*  <  0.05). On the other hand, a comparatively stronger correlation was found between the amikacin resistance phenotype and *aaC4* among the strains (*p*  = 0.043), while there was also a significant differential correlation between the tetracycline resistance phenotype and the *tet*C resistance gene (*p* = 0.006).

### 3.5. Detection of Class 1 Integrons

Class 1 integrons were detected in 42 of the 135 *E. coli* strains (31.1%). Detailed information on the *E. coli* strains, including ID number, phylogenetic cluster, resistance profiles, drug resistance profiles, class 1 integrons, and virulence genes, is shown in a Supplementary Table S1.

### 3.6. Distribution of Virulence Genes in E. coli Strains

**Figure 3:**
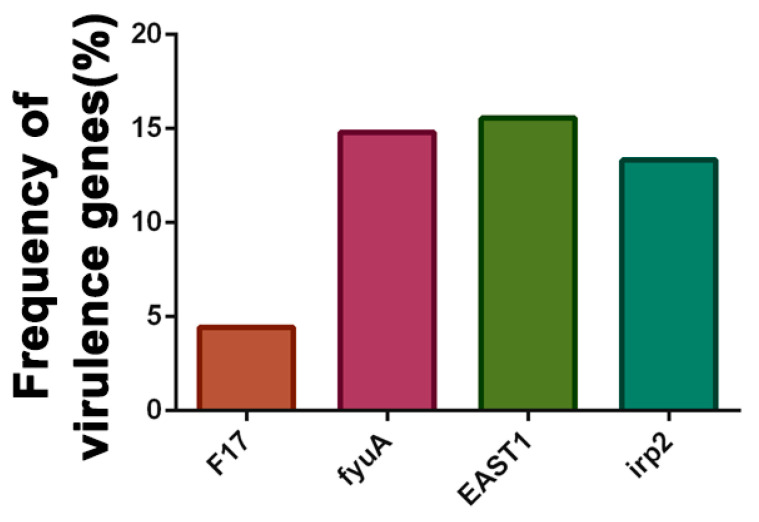
Distribution of virulence genes in *E. coli* strains.

The identification of 14 virulence genes in *E. coli* strains is shown in Figure 3. The most prevalent virulence gene was *fyu*A (14.81%), the marker gene of the iron scavenger receptor, followed by *EAST1* (15.55%) and *irp2* (13.33%). Notably, the gene coding for *hly*A, *Stx1*, *Stx2*, *K99*, *987p*, *LT*, *F18*, and *CS31A* was not detected in any strain.

### 3.7. Biofilm Formation Ability and Motility Ability of the 21EC78 Strain

**Figure 4:**
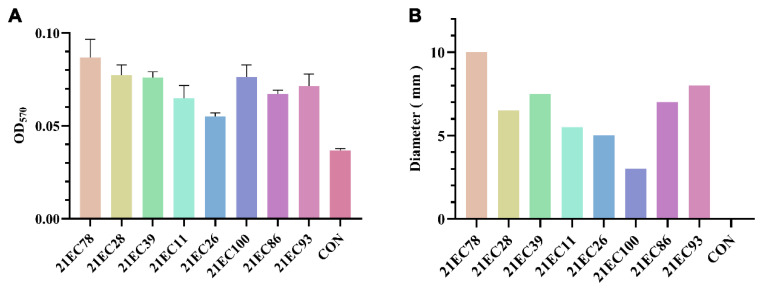
The determination of biofilm formation ability and motility ability of strain 21EC78. (**A**) Comparison of biofilm formation ability of eight strains of *E. coli*. (**B**) Comparison of motion ring diameter of eight strains of *E. coli*.

A biofilm, which is formed by bacteria to adapt to their natural environment, can enhance bacterial resistance and enable them to withstand external environmental stress. The ability of different *E. coli* strains to form a biofilm was initially assessed, and the results are shown in Figure 4A. Various clinical strains exhibited varying abilities to form biofilms, with strain 21EC78 demonstrating the most robust biofilm-forming capacity. In addition, the results of the motility assay are shown in Figure 4B, where the motility ring of 21EC78 on the semi-solid flat dish was significantly larger than that of the other tested strains. We, therefore, chose strain 21EC78 for further investigation.

### 3.8. Characterization of Resistance Profiles, Phylogrouping, Antimicrobial Resistance Genes, and Virulence Genes of the 21EC78 Strain

The antimicrobial susceptibility analysis showed that 21EC78 was an MDR strain with the resistance profile of CTRX-CAZ-AML-AM-TET-DOX-ENR-OFX-AMK-AZI-SXT-SMM. According to the genomic similarity analysis using phylogrouping, 21EC78 belongs to group B2. The WGS results of 21EC78 revealed that it carries 11 resistance genes—*sul1*, *sul2*, *dfrA17*, *aph(6)-Id*, *aph(3″)-Ib*, *aad*A5, *mph*(A), *sit*ABCD, *tet*(A), *bla*TEM-1B, *qac*E—and also 16 virulence genes—*afa*D, *cia*, *cma*, *cva*C, *gad*, *hly*E, *hly*F, *iro*N, *iss*, *iuc*C, *iut*A, *lpf*A, *omp*T, *sit*A, *ter*C, *tra*T. Notably, the ST type of 21EC78 is ST1196, the phylogroup belongs to *fim*H31, and the serotype is O83:H28.

### 3.9. Differences in the Pathogenicity of Challenge Methods to SPF Chickens

The chickens in the intraperitoneal injection group had a mortality rate of 100% within two days. Conversely, none of the chickens in the oral group died within seven days and exhibited no other clinical signs, indicating good health and spirits (Table 4). The results showed that the pathogenicity of strain 21EC78 was greater when administered through intraperitoneal injection compared to the oral route.

### 3.10. Differences in the Pathogenicity to Chicken Breeds

The mortality rate was significantly lower in the Luhua chickens compared to the SPF chickens (Table 4). The results indicated that the 21EC78 strain exhibited greater pathogenicity in SPF chickens compared to Luhua chickens.

### 3.11. Discussion

*E. coli* is a member of the Enterobacteriaceae family, a common bacterium that lives in the intestinal tract of humans and animals. While most of these bacteria are harmless, when the body is in a state of hypoimmunity or stress, some strains can cause gastrointestinal infections, secondary infections, and systemic diseases in animals and humans. These upsets can result not only in serious economic losses to the poultry industry but can also cause great harm to human health and food safety.

Antimicrobial resistance is one of the most critical threats to human health in recent years and, in the future, is especially associated with zoonotic pathogens [47]. Our study assessed the prevalence of resistance to common antimicrobial agents, as well as the diversity and distribution of resistance genes and virulence genes in *E. coli* strains obtained from a white feather broiler farm.

*E. coli* can be mainly divided into seven phylogenetic types: A, B1, B2, C, D, F, and clade I [20]. Among them, groups B2 and D are mainly extraintestinal infection and invasive strains, most of them pathogenic *E. coli*, which can cause urinary tract infections of the body and have a strong invasive ability [48]. The *E. coli* strained in this study was predominantly classified as group B1 (37.04%), while the isolation rates of groups B2 and D were considerably lower, at 2.22% and 11.11%, respectively. In addition, this evolutionary group analysis approach may be slightly constrained by evolutionary lineages and interactions, while integration into more complete phylogenetic groups would require more precise methods such as MLST (multilocus-sequence typing) [21].

Among the 15 antibiotics used in this study, all *E. coli* strains were resistant to at least five types of antibiotics, which is similar to the results of a study by Zhang et al. who strained more than 95% of *E. coli* as multidrug-resistant bacteria in marketed retail food in 39 cities in China [49]. Moreover, alarming MDR has also been observed in previous studies from broiler chicken farms in other countries [50,51,52]. Therefore, there is a need for increased surveillance of antimicrobial resistance in *E. coli* to prevent the further spread of multidrug-resistant *E. coli*. In this study, the resistance rates of 135 strains of *E. coli* to doxycycline, tetracycline, cotrimoxazole, and ampicillin were 97.78%, 97.78%, 96.30%, and 95.56%, respectively. It is worth noting that no drug was found to be susceptible to all strained strains. It is crucial to prioritize the rational and cautious utilization of these potent and wide-ranging antibiotics in clinical practice. The high MDR rates observed in the current study signify that the current therapeutic strategies for *E. coli* infections in intensive poultry farms are restricted; thus, it is highly recommended that significant measures be taken to manage potential risks from poultry farms, such as by reducing and regulating antibiotic application. Furthermore, the extensive spread of MDR *E. coli* in food animals, particularly in broilers, presents a food security concern and a major threat to public health.

An important reason for bacterial resistance to antibiotics is the presence of associated resistance genes, and many studies have shown that MDR is associated with bacteria carrying resistance genes [2]. As previously mentioned, the *blaCTX-M* gene is the most common extended-spectrum β-lactamases (ESBL)-encoding gene in humans and animals [53,54]. In this study, the prevalence of *blaCTX-M* was over 85%, which is much higher than the prevalence found in other studies. In addition, we found a prevalence of *blaTEM* of 96.30% in this study, which is similar to previous reports in *E. coli* from food-producing animals [55]. In addition, the *qnr*S gene was also detected, which was the most prevalent PMQR gene among ESBL-producing Enterobacter species strains from individuals in China [56]. These findings might indicate that the *qnr*S gene is locally disseminated among food-producing animals and human beings in China. By analyzing the correlation between the genotypic–phenotypic antimicrobial resistance of the strains, a relatively stronger correlation was revealed between the ceftazidime and cefotaxime resistance phenotypes and *bla*CTX-M among the strains. However, there was no correlation between the ceftriaxone resistance phenotype and *bla*CTX-M, which is inconsistent with the relatively strong correlation found between ceftriaxone and *bla*CTX-M found in companion animal-derived strains by Cui et al. [9]. This inconsistency is possibly due to differences in animals carrying resistance genes from different sources. Conversely, a notable absence of correlation was observed between the resistance phenotypes and the resistance genes for sulfonamides and polymyxins, likely due to the intricate mechanisms of bacterial resistance to these drug classes.

Among the 135 *E. coli* strains, the positive rates of *E. coli* carrying *irp2*, *fyu*A, *EAST1*, and *F17* were 13.33%, 14.81%, 14.07%, and 4.44%, respectively. The *EAST1* gene was originally identified in strains of an enteroaggregative *E. coli* (EAEC) strained from the stool of a Chilean child with diarrhea [57]. Several studies have shown that the gene encoding *EAST1* is not restricted to EAEC and is widely distributed among DEC pathotypes and other human enteric pathogens [58,59,60]. The *irp2* gene mediates the iron uptake system of highly pathogenic strains and is associated with *E. coli* virulence [61] and has been detected in human pathogenic *E. coli* strains [62,63]. The *irp2* gene is located on a pathogenicity island that has spread between *E. coli* species by horizontal gene transfer [64]. The *fyu*A gene is highly pathogenic and is often used as an indicator for the presence or absence of high-pathogenicity islands [65].

*E. coli* often forms biofilms to resist oxidative stress and damage caused by some antimicrobial drugs [66]. In this study, an *E. coli* strain called 21EC78 with strong biofilm-forming ability was strained. The drug susceptibility test revealed that it had multidrug resistance. The WGS showed that it had 16 virulence genes and 11 drug resistance genes. In addition, the pathogenicity of 21EC78 in different chicken breeds showed that Luhua chickens exhibited a comparatively lower frequency of occurrence and mortality in contrast to SPF chickens. We also investigated the pathogenicity of 21EC78 to SPF chickens through oral and intraperitoneal injection challenge methods. The results showed that SPF chickens in the oral administration group did not die, regardless of the challenge dose, while SPF chickens in the intraperitoneal injection group had a 100% mortality rate within three days. The multidrug-resistant *E. coli* 21EC78 strain, which carries multiple resistance and virulence genes, was strained from a broiler farm and can be transmitted to humans through the food chain, suggesting a risk of cross-species transmission. Furthermore, pathogenicity assays demonstrated its high virulence toward chickens, which warrants our attention.

In recent years, the problem of the drug resistance of pathogenic *E. coli* strains has become more serious with the increasing intensification of agricultural practices [67]. There is an urgent need for appropriate prevention and control measures. Hence, it is imperative to conduct epidemiological investigation and monitor drug resistance genes in *E. coli* to provide a scientific foundation for the control and clinical management of drug-resistant strains.

## 4. Conclusions

In this study, we investigated and analyzed antibiotic resistance, class 1 integrons, resistance genes, virulence genes, and phylogenetic subgroups of *E. coli* strains from broiler farms. The isolated strain, called 21EC78, with extremely strong membrane-forming ability, was found to have strong pathogenicity to SPF chickens. It is worth studying, in the future, more broiler farms, undergoing long-term monitoring of *E. coli*.

## Figures and Tables

**Table 1:** Proportion of phylogenetic clusters of *E. coli* strains.

Phylogenetic Cluster	Proportion
A	11.11% (15/135)
B1	37.04% (50/135)
B2	2.22% (3/135)
D	11.11% (15/135)
E	11.85% (16/135)
A or C	20.74% (28/135)
Clade Ⅰ	1.48% (2/135)
F	4.44% (6/135)

**Table 2:** The drug resistance profiles and proportion of *E. coli* strains.

Type of Drug Resistance	Drug Resistance Profile	Proportion	All
5	DOX-AMK-AZI-SXT-SMM	0.7% (1/135)	0.7% (1/135)
6	TET-DOX-AMK-SXT-SMM-PB	0.7% (1/135)	4.44% (6/135)
	AML-AM-TET-DOX-SXT-SMM	2.2% (3/135)	
	TET-DOX-ENR-SXT-SMM-PB	0.7% (1/135)	
	AML-AM-TET-DOX-SMM-PB	0.7% (1/135)	
7	AML-AM-TET-DOX-ENR-SXT-SMM	2.2% (3/135)	4.44% (6/135)
	AML-AM-TET-DOX-SXT-SMM-PB	2.2% (3/135)	
8	AML-AM-ENR-OFX-AZI-SXT-SMM-PB	0.7% (1/135)	15.56% (21/135)
	AML-AM-TET-DOX-ENR-OFX-SXT-SMM	7.40% (10/135)	
	AML-AM-TET-DOX-ENR-SXT-SMM-PB	2.96% (4/135)	
	TET-DOX-ENR-OFX-AMK-AZI-SXT-SMM	0.7% (1/135)	
	TET-DOX-ENR-AMK-AZI-SXT-SMM-PB	0.7% (1/135)	
	AML-AM-TET-DOX-ENR-OFX-SMM-PB	0.7% (1/135)	
	AML-AM-TET-DOX-AMK-SXT-SMM-PB	0.7% (1/135)	
	CTRX-CAZ-AML-AM-TET-DOX-ENR-OFX	0.7% (1/135)	
	CTRX-AML-AM-TET-DOX-ENR-SXT-SMM	0.7% (1/135)	
9	CTRX-CAZ-CTX-AML-AM-TET-DOX-SXT-SMM	8.89% (12/135)	20.74% (28/135)
	CTRZ-AML-AM-TET-DOX-ENR-OFX-SXT-SMM	1.48% (2/135)	
	AML-AM-TET-DOX-ENR-AMK-AZI-SXT-SMM	0.7% (1/135)	
	AML-AM-TET-DOX-ENR-OFX-AZI-SXT-SMM	1.48% (2/135)	
	AML-AM-TET-DOX-ENR-OFX-SXT-SMM-PB	2.2% (3/135)	
	CTRX-CAZ-AML-AM-TET-DOX-ENR-SXT-SMM	1.5% (2/135)	
	CAZ-AML-AM-TET-DOX-ENR-SXT-SMM-PB	0.7% (1/135)	
	CAZ-AML-AM-TET-DOX-ENR-OFX-SXT-SMM	3.70% (5/135)	
10	CTRX-CAZ-CTX-AML-AM-TET-DOX-ENR-SXT-SMM	9.63% (13/135)	23.70% (32/135)
	CAZ-CTX-AML-AM-TET-DOX-ENR-AZI-SXT-SMM	0.7% (1/135)	
	AML-AM-TET-DOX-ENR-OFX-AMK-AZI-SXT-SMM	4.4% (6/135)	
	AML-AM-TET-DOX-ENR-OFX-AMK-SXT-SMM-PB	0.7% (1/135)	
	AML-AM-MEM-TET-DOX-ENR-OFX-SXT-SMM-PB	0.7% (1/135)	
	CTRX-CTX-AML-AM-TET-DOX-ENR-OFX-SXT-SMM	0.7% (1/135)	
	AML-AM-TET-DOX-ENR-AMK-AZI-SXT-SMM-PB	0.7% (1/135)	
	CTRX-AML-AM-TET-DOX-ENR-OFX-SXT-SMM-PB	1.5% (2/135)	
	CTRX-AML-AM-TET-DOX-ENR-OFX-AZI-SXT-SMM	0.7% (1/135)	
	CTRX-CAZ-AML-AM-TET-DOX-ENR-OFX-SXT-SMM	0.7% (1/135)	
	CAZ-AML-AM-TET-DOX-ENR-OFX-SXT-SMM-PB	3.0%(4/135)	
11	CTRX-CAZ-CTX-AML-AM-TET-DOX-ENR-OFX-SXT-SMM	2.2% (3/135)	14.81% (20/135)
	CTRX-CAZ-CTX-AML-AM-TET-DOX-ENR-AZI-SXT-SMM	1.5% (2/135)	
	AML-AM-TET-DOX-ENR-OFX-AMK-AZI-SXT-SMM-PB	3.7% (5/135)	
	CTRX-CTX-AML-AM-TET-DOX-ENR-OFX-AMK-AZI-PB	0.7% (1/135)	
	CTRX-CTX-AML-AM-TET-DOX-ENR-OFX-AMK-SXT-SMM	0.7% (1/135)	
	CTRX-AML-AM-TET-DOX-ENR-OFX-AMK-AZI-SXT-SMM	1.5% (2/135)	
	CTRX-CTX-AML-AM-TET-DOX-ENR-OFX-AZI-SXT-SMM	0.7% (1/135)	
	CTRX-CAZ-CTX-AML-AM-TET-DOX-ENR-SXT-SMM-PB	0.7% (1/135)	
	CTRX-CAZ-AML-AM-TET-DOX-ENR-OFX-SXT-SMM-PB	2.2% (3/135)	
	CTRX-CAZ-AML-AM-TET-DOX-ENR-OFX-AZI-SXT-SMM	0.7% (1/135)	
12	CTRX-CAZ-CTX-AML-AM-TET-DOX-ENR-OFX-AZI-SXT-SMM	6.7% (9/135)	10.37% (14/135)
	CTRX-CAZ-CTX-AML-AM-TET-DOX-ENR-OFX-AMK-SXT-SMM	0.7% (1/135)	
	CTRX-CAZ-AML-AM-TET-DOX-ENR-OFX-AMK-AZI-SXT-SMM	0.7% (1/135)	
	CTRX-CTX-AML-AM-TET-DOX-ENR-OFX-AMK-SXT-SMM-PB	0.7% (1/135)	
	CTRX-CAZ-CTX-AML-AM-TET-DOX-ENR-OFX-SXT-SMM-PB	1.5% (2/135)	
13	CTRX-CTX-AML-AM-TET-DOX-ENR-OFX-AMK-AZI-SXT-SMM-PB	0.7% (1/135)	3.7% (5/135)
	CTRX-CAZ-CTX-AML-AM-TET-DOX-ENR-OFX-AZI-SXT-SMM-PB	0.7% (1/135)	
	CTRX-CAZ-CTX-AML-AM-TET-DOX-ENR-OFX-AMK-AZI-SXT-SMM	1.5% (2/135)	
	CTRX-CAZ-CTX-AML-AM-TET-DOX-ENR-OFX-AMK-SXT-SMM-PB	0.7% (1/135)	
14	CTRX-CAZ-CTX-AML-AM-TET-DOX-ENR-OFX-AMK-AZI-SXT-SMM-PB	0.7% (1/135)	1.5% (2/135)
	CTRX-CAZ-CTX-AML-AM-MEM-TET-DOX-ENR-OFX-AMK-AZI-SXT-SMM	0.7% (1/135)	

**Table 3:** Correlation matrix between resistance phenotype and genotype.

Antibiotics Resistance Phenotype	Resistance Genes	Characteristics of Strains				*p* Value
		P−/G+	P+/G−	P+/G+	P−/G−	
CTRX	*bla*TEM	61	3	69	2	0.761
	*bla*CTX-M	54	1	71	9	0.004 **
CAZ	*bla*TEM	64	3	66	2	0.685
	*bla*CTX-M	58	2	67	8	0.041 *
CTX	*bla*TEM	77	3	53	2	0.392
	*bla*CTX-M	69	0	56	10	0.006 **
ENR	*qnr*S	22	3	110	0	0.442
	*oqx*A	1	91	22	21	0.088
OFX	*qnr*S	53	3	79	0	0.159
	*oqx*A	7	66	16	46	0.341
AMK	*aaC2*	37	25	5	68	0.053
	*aaC4*	67	5	25	38	0.043 *
TET	*tet*A	2	5	128	0	0.83
	*tet*B	2	4	129	0	0.803
	*tet*C	1	132	1	1	0.006 **
DOX	*tet*A	1	5	129	0	0.844
	*tet*B	1	4	130	0	0.861
	*tet*C	0	133	1	1	0.931
SXT	*sul1*	1	109	22	3	0.667
	*sul2*	1	124	7	3	0.128
SMM	*sul1*	1	110	22	2	0.448
	*sul2*	0	124	8	3	0.638
PB	*mcr-1*	2	43	2	88	0.473

P−/G+: the number of phenotypic susceptible strains (P−) with resistance genes (G+) for antimicrobial identified. P+/G−: the number of phenotypic resistance strains (P+) with no resistance gene (G−) for the antimicrobial identified. P+/G+: the number of P+ with G+ for antimicrobial identified. P−/G−: the number of P− with G− for the antimicrobial identified. *p* values: * *p* < 0.05; ** *p* < 0.01.

**Table 4:** Pathogenicity of *E. coli* strain 21EC78 in challenge experiment with SPF and Luhua chickens.

Group	No. of Chicken	Chicken Species	Treatment	Dose (CFU)	Death Rate (%)
A	6	SPF	oral administration	2 × 10^6^	0
B	6			2 × 10^7^	0
C	6			2 × 10^8^	0
D	6			0	0
E	6		intraperitoneal injection	2 × 10^5^	100
F	6			2 × 10^6^	100
G	6			2 × 10^7^	100
H	6			2 × 10^8^	100
I	6			2 × 10^9^	100
J	6			0	0
K	6	Luhua	intraperitoneal injection	2 × 10^5^	0
L	6			2 × 10^6^	0
M	6			2 × 10^7^	0
N	6			2 × 10^8^	66.7
O	6			2 × 10^9^	100
P	6			0	0

## Data Availability

The original contributions presented in the study are included in the article/Supplementary Material; further inquiries can be directed to the corresponding author(s).

## Supplementary Materials

The following supporting information can be downloaded at: https://www.mdpi.com/article/10.3390/microorganisms11122939/s1, Table S1: Detailed information on the *E. coli* strains.

## Abbreviations

*E. coli*: *Escherichia coli*; WGS: whole-genome sequencing; MDR: The multidrug-resistant; MLST: multilocus sequence typing; AM: ampicillin; AMX: amoxicillin; SXT: trimethoprim-sulfamethoxazole; TET: tetracycline; MEM: meropenem; ENR: enrofloxacin; CTRX: ceftriaxone; DOX: doxycycline; CTX: cefotaxime; CAZ: ceftazidime; OFX: ofloxacin; AMK: amikacin; AZI: azithromycin; SMM: sulfamonomethoxine; PB: polymyxin B.
